# Early-Stage Luminal B-like Breast Cancer Exhibits a More Immunosuppressive Tumor Microenvironment than Luminal A-like Breast Cancer

**DOI:** 10.3390/biom15010078

**Published:** 2025-01-07

**Authors:** Tânia Moura, Olga Caramelo, Isabel Silva, Sandra Silva, Manuela Gonçalo, Maria Antónia Portilha, João N. Moreira, Ana M. Gil, Paula Laranjeira, Artur Paiva

**Affiliations:** 1 Flow Cytometry Unit, Department of Clinical Pathology, Hospitais da Universidade de Coimbra, Unidade Local de Saúde de Coimbra, Avenida Bissaya Barreto, Bloco Hospitalar de Celas, nº 205, 3000-076 Coimbra, Portugal; tania.moura@ua.pt (T.M.); 14546@ulscoimbra.min-saude.pt (I.S.); 9656@ulscoimbra.min-saude.pt (S.S.); 2 Department of Chemistry, University of Aveiro, Campus Universitário de Santiago, 3810-193 Aveiro, Portugal; agil@ua.pt; 3 Gynecology Department, Hospitais da Universidade de Coimbra, Unidade Local de Saúde de Coimbra, Praceta Prof. Mota Pinto, 3000-075 Coimbra, Portugal; olgalgcaramelo@gmail.com; 4 Medical Imaging Department, Hospitais da Universidade de Coimbra, Unidade Local de Saúde de Coimbra, Praceta Prof. Mota Pinto, 3000-075 Coimbra, Portugal; manuela_goncalo@hotmail.com (M.G.); mafportilha@gmail.com (M.A.P.); 5 Center for Neuroscience and Cell Biology (CNC), University of Coimbra, 3004-504 Coimbra, Portugal; jmoreira@ff.uc.pt; 6 Center for Innovative Biomedicine and Biotechnology (CIBB), University of Coimbra, 3004-504 Coimbra, Portugal; 7 University of Coimbra, CIBB, Faculty of Pharmacy, Pólo das Ciências da Saúde, Azinhaga de Santa Comba, 3000-548 Coimbra, Portugal; 8 CICECO─Aveiro Institute of Materials, Department of Chemistry, University of Aveiro, Campus Universitário de Santiago, 3810-193 Aveiro, Portugal; 9 Coimbra Institute for Clinical and Biomedical Research (iCBR), Group of Environmental Genetics of Oncobiology (CIMAGO), Faculty of Medicine (FMUC), University of Coimbra, Azinhaga de Santa Comba, 3000-548 Coimbra, Portugal; 10 Center for Innovative Biomedicine and Biotechnology (CIBB), University of Coimbra, Pólo das Ciências da Saúde, Azinhaga de Santa Comba, 3000-504 Coimbra, Portugal; 11 Clinical Academic Center of Coimbra (CACC), 3000-061 Coimbra, Portugal; 12 Faculty of Medicine, University of Coimbra, Polo das Ciências da Saúde, Sub-Unidade 1, Azinhaga de Santa Comba, Celas, 3000-548 Coimbra, Portugal; 13 Instituto Politécnico de Coimbra, ESTESC—Coimbra Health School, Ciências Biomédicas Laboratoriais, Rua 5 de Outubro, 3046-854 Coimbra, Portugal

**Keywords:** breast cancer, tumor microenvironment, immune cells, monocytes, T cells

## Abstract

Background: Breast cancer is a heterogeneous malignant disease with a varying prognosis and is classified into four molecular subtypes. It remains one of the most prevalent cancers globally, with the tumor microenvironment playing a critical role in disease progression and patient outcomes. Methods: This study evaluated tumor samples from 40 female patients with luminal A and B breast cancer, utilizing flow cytometry to phenotypically characterize the immune cells and tumor cells present within the tumor tissue. Results: The luminal B-like tumor samples exhibited increased infiltration of CD4^+^ cells, regulatory T cells (Tregs), and Th17 cells and decreased levels of NK cells, γδ T cells, Th1 cells, and follicular T cells, which is indicative of a more immunosuppressive tumor microenvironment. Conclusions: These findings suggest that luminal B-like tumors have a microenvironment that is less supportive of effective anti-tumor immune responses compared to luminal A tumors. This study enhances the understanding of the immunological differences between luminal subtypes of breast cancer and identifies potential new therapeutic targets and biomarkers that could drive advancements in precision medicine for breast cancer management.

## 1. Introduction

Breast cancer is the most prevalent neoplasm among women worldwide and remains a leading cause of cancer-related deaths, with over 2.3 million new cases diagnosed in 2020; in the same year, it contributed to nearly 12% of all new cases and led to 685,000 deaths [1]. This high incidence underscores the urgent need to better understand the mechanisms underlying the development and progression of this cancer and to improve diagnostic and therapeutic strategies. A major challenge in breast cancer management is its significant heterogeneity, as tumors can vary widely in histological, genetic, and molecular characteristics. Based on molecular profiling, breast cancer is categorized into four main subtypes: luminal A-like, luminal B-like, HER^2+^, and triple negative [2,3,4]. These subtypes not only differ in biological behavior, but also in their responses to treatments and prognoses, with luminal subtypes generally associated with more favorable clinical outcomes [3]. Luminal A-like tumors are typically low grade, strongly positive for estrogen receptor (ER) and progesterone receptor (PgR), and negative for human epidermal growth factor receptor 2 (HER2). They also exhibit low proliferation. Luminal B-like tumors are hormone receptor (HR)-positive but may have variable degrees of ER and PgR expression; they are higher grade and have higher proliferation than luminal A-like tumors. HR-positive and HER2-negative tumors are the most common type of early breast cancer, accounting for over 70% of all cases worldwide [5].

Despite the advances in therapeutic options, including surgery, chemotherapy, radiotherapy, hormonal therapies, and immunotherapy, breast cancer remains a formidable challenge due to the variability in therapeutic responses, recurrence rates, and the propensity for metastasis [3,6]. Metastasis is the leading cause of mortality in breast cancer, with the most common metastatic sites being bones, lungs, liver, and brain [7]. The metastatic potential of a tumor depends on the intricate interactions between cancer cells and the cells contained within the tumor microenvironment (TME) [8]. This microenvironment includes a diverse array of cells, such as immune cells, fibroblasts, endothelial cells, and components of the extracellular matrix [9,10].

Within the TME, immune cells play crucial roles in either promoting or inhibiting tumor progression. T cells, as key players in the adaptive immune response, exhibit contrasting effects within the tumor context [11,12]. Cytotoxic (CD8^+^) T cells are known for their ability to target and destroy tumor cells [13]. However, other T cell subtypes, such as regulatory T cells (Tregs) and Th17 cells, can suppress the immune response against the malignant cells, thereby facilitating tumor progression [14,15]. An increase in these suppressive mechanisms, namely the higher infiltration of regulatory immune cells within the TME, is often linked to poorer outcomes across various cancers, including breast cancer [7,16,17,18].

Programmed cell death ligand 1 (PD-L1), also referred to as CD274, is a transmembrane protein that is often expressed on tumor cells, where it interacts with the PD-1 receptor on T cells, leading to immune suppression. In breast cancer, elevated PD-L1 expression is associated with poor prognosis and is observed primarily in tumor cells and immunosuppressive cells [19,20,21]. PD-1 acts as an inhibitor of both adaptative and innate immune responses. It is expressed on activated T cells, natural killer (NK) cells and B lymphocytes, macrophages, dendritic cells (DCs), and monocytes [22]. Similarly, CD206, also known as the mannose receptor C type 1 (MRC1), is predominantly expressed on M2 macrophages and has been linked to tumor-promoting activities such as angiogenesis and metastasis [23,24].

While the presence of immune cells in the TME generally correlates with improved clinical outcomes, the composition and functional roles of these cells can vary significantly between breast cancer subtypes. This highlights the need to further explore the phenotypic characteristics of immune cells within the TME. This study aims to identify and phenotypically characterize immune cell populations in breast tissue samples, using flow cytometry. By examining differences in T cell subpopulations between luminal subtypes, the study seeks to provide new insights into the role of the immune microenvironment in breast cancer progression, identify potential prognostic and therapeutic biomarkers, and support the development of more personalized and effective treatment strategies. Additionally, this deeper understanding can potentially enhance the precision in differentiating luminal subtypes, ultimately contributing to improved patient stratification and outcomes.

## 2. Materials and Methods

### 2.1. Clinicopathological Characteristics of the Patients

Tumor samples from 40 women with breast carcinoma (mean age: 59 ± 7) were collected at the Genecology Department of Hospitais da Universidade de Coimbra, Unidade Local de Saúde de Coimbra, Portugal. The biological material was obtained through ultrasound-guided breast biopsy and subsequently sent to the Flow Cytometry Unit of Hospitais da Universidade de Coimbra, Unidade Local de Saúde de Coimbra, Portugal, for analysis. All the experimental protocols adhered to ethical standards and were previously approved by the Ethics Committee of Unidade Local de Saúde de Coimbra (reference number 017/24 CE, 2 February 2024).

The clinicopathological classification of tumors was carried out according to the expression of hormone receptors (estrogen receptor (ER) and progesterone receptor (PR)) and HER2, as well as the assessment of the Ki-67 proliferation index. The samples were classified into the following categories: luminal A (ER+ and/or PR+, Ki-67 < 20%) and luminal B (ER+ and/or PR+, Ki-67 ≥ 20%). Breast carcinoma staging was conducted according to the guidelines of the eighth edition of the TNM system, as established by the American Joint Committee on Cancer (AJCC) Cancer Staging System [25]. The clinical and demographic characteristics of the patients are detailed in Table 1.

### 2.2. Phenotypic Characterization of Cells Obtained from the Breast Cancer Biopsy

Tumor samples, collected into a flask containing phosphate-buffered saline (PBS), were first subjected to multiple PBS washes to release the maximum cellular content. The dissociation of the tumor tissue was performed using only mechanical dissociation. The resulting cell suspension was transferred to a Falcon tube and centrifuged at 540× *g* for 5 min at room temperature. To identify the different immune subpopulations, two separate monoclonal antibody panels were employed: an 8-color panel for monocyte/macrophage characterization and a 12-color panel for the study of T lymphocytes (Table 2). Antibodies were added to cells suspended in a final volume of 300 μL and incubated in the dark at room temperature for 10 min. Then, 2 mL of FACSLysing solution (BD, Becton Dickinson Biosciences, San Jose, CA, USA) was added, and the samples were incubated again in the dark for an additional 10 min.

The samples were subsequently centrifuged at 540× *g* for 5 min to remove the supernatant. The resulting cell pellet was washed with PBS, resuspended in 500 μL of PBS, and immediately acquired in the flow cytometer.

### 2.3. Data Acquisition

For the 8-color antibody panel, samples were acquired using the cytometer FACSCanto II flow cytometer (BD, San Jose, CA, USA), equipped with FACSDiva software (V.6.1.2; BD), while the cytometer FACSLyric (Becton Dickinson Biosciences, San Jose, CA, USA), equipped with FACSuite software (V1.5.0.925; BD), was used for the 12-color panel. The samples were analyzed using instrument settings that were standardized and recommended by the Euroflow consortium [26,27]. The analysis of the data was processed using Infinicyt™ software (V.2.05; Cytognos SL, Salamanca, Spain).

### 2.4. Gating Strategy

**Figure 1:**
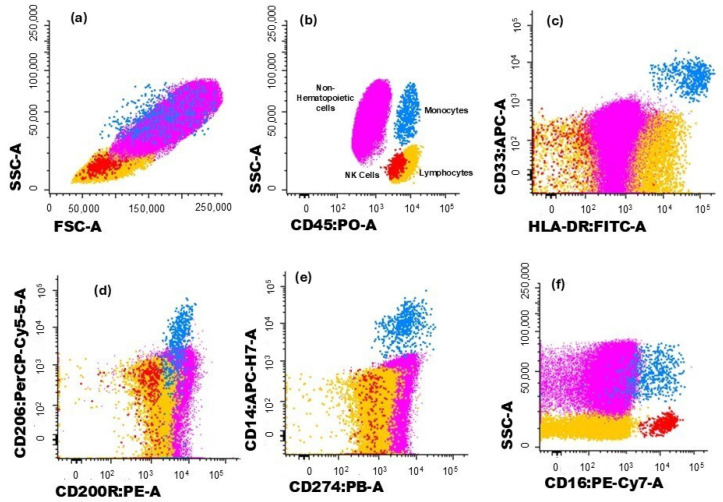
Dot plot histograms illustrate the gating strategy for identifying immune cells infiltrating the tumor. Breast cancer cells are represented in pink, monocytes in blue, and NK cells in red; the remaining cells are in yellow. (**a**) Cells were identified based on typical FSC and SSC characteristics. (**b**) Hematopoietic cells were identified by the expression of CD45, while non-hematopoietic cells (in pink) correspond to CD45^−^ events. (**c**) Monocytes (blue) were identified through the expression CD33 and HLA-DR. The expression of (**d**) CD200R and (**e**) CD206 was assessed in monocytes. (**f**) The expression of CD274 was verified in monocytes and non-hematopoietic cells.

**Figure 2:**
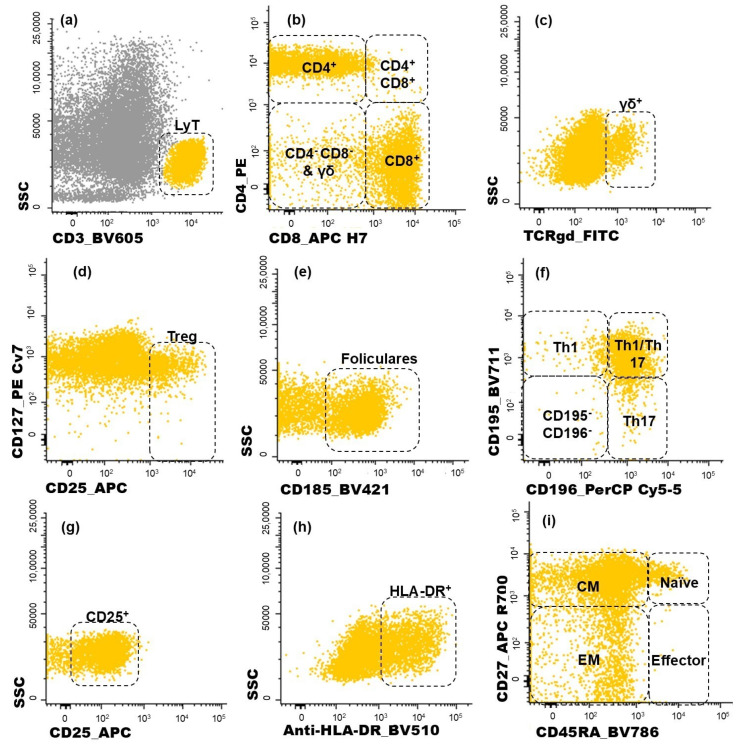
Dot plot histograms illustrating the gating strategy used for identifying CD4^+^ T cell subpopulations infiltrating the tumor. T lymphocytes recognized through the expression of CD3 (**a**). Within this population, five subgroups were identified (CD4^+^, CD8^+^, CD4^+^CD8^+^, CD4^−^CD8^−^, γδ^+^ T cells) (**b**,**c**); regulatory T cells (Treg) were characterized as CD25^bright^ CD127^−/low^ (**d**); the follicular-like T cells correspond to CD185-positive cells (**e**). CD4 T cells were further characterized as Th1 (CD195^+^CD196^−^), Th17 (CD195^−^CD196^+^), Th1/17 (CD195^+^CD196^+^), and, lastly, CD195^−^CD196^−^ CD4 T cells (**f**); the activated (CD25^+^ or HLA-DR^+^) subsets of T cells were measured in all T cell subsets identified (**g**,**h**); CD45RA and CD27 were used to identify naïve (CD45RA^+^ CD27^+^), central memory (CM, CD45RA^−^ CD27^+^), effector memory (EM, CD45RA^−^ CD27^−^), and terminal effector (CD45RA^+^ CD27^−^) maturation-associated T cell compartments on the abovementioned subsets of T cells (**i**). Yellow events represent T cells, and grey represents other cells.

The gating strategy used to characterize the different immune cell populations analyzed in this study is illustrated in Figure 1 and Figure 2. Hematopoietic cells were identified by their positivity for CD45. Non-hematopoietic cells (corresponding to breast tumor cells) were defined by the absence of CD45, along with their forward scatter (FSC) and side scatter (SSC) light dispersion properties, and the expression of cytokeratin 18 and CD326 (EpCAM) (Figure 1a,b).

Monocytes were identified through typical FSC and SSC characteristics, intermedium expression of CD45, and high expression of CD33 and HLA-DR (Figure 1c). The expressions of CD200R (Figure 1d), CD206 (Figure 1d), and CD274 (Figure 1e) were further assessed for monocytes, and CD200R and CD274 expressions were assessed in non-hematopoietic cells.

Lymphocytes were identified by the high expression of CD45 and low FSC and SSC and, within this cell population, NK cells were recognized by the expression of CD16 (Figure 1f). On the other hand, T cells were identified based on their positivity for CD3 (Figure 2a). T cell subsets were further identified (Figure 2b,c): CD4^+^ T cells (distinguished by the presence of CD4 and the absence of CD8), CD8^+^ T cells (characterized by CD8 expression and the lack of CD4), CD4^+^CD8^+^ T cells (those co-expressing CD4 and CD8), CD4^−^CD8^−^ T cells (presenting the CD3^+^CD4^−^CD8^−^γδ^−^ phenotype), and γδ T cells (defined by the presence of the TCR γδ).

Among the CD4^+^ T cells, regulatory T (Treg) cells were identified by the combined bright expression of CD25 and low-to-negative expression of CD127 (Figure 2d). Follicular T cells were characterized by CD185 expression (Figure 2e). Additionally, the sense of CD4^+^ T cell polarization was evaluated based on CD195 and CD196 expression (Figure 2f), allowing the identification of the following functional subsets: Th1 (CD195^+^CD196^−^), Th17 (CD195^−^CD196^+^), and Th1/17 (CD195^+^CD196^+^) cells. The remaining CD195^−^CD196^−^ CD4^+^ T cells correspond to a mixture of other functional subsets and include Th0 cells.

The same approach used to identify the subpopulations of CD4^+^ T cells was applied to CD8^+^ T cells. For each one of the subsets identified among the CD4^+^ T and CD8^+^ T cells, the proportion of activated cells was determined by analyzing the intermediate activation marker CD25 (Figure 2g) and late activation marker HLA-DR (Figure 2h). Additionally, the functional compartments of T cells were assessed for CD4^+^ and CD8^+^ T cells, as depicted in Figure 2i. Naïve T cells were defined by the expression of CD45RA and CD27. Central memory (CM) T cells, which lack CD45RA but remain CD27-positive, were distinguished from effector memory (EM) T cells, characterized by the absence of both CD4RA and CD27. Terminal effector cells were identified by the expression of CD45RA in the absence of CD27.

### 2.5. Statistical Analysis

The statistical analysis of the results was performed using GraphPad Prism 8 software (San Diego, CA, USA, Version 8.0.1). Initially, the clinicopathological characteristics of the tumors, including histological grade, nodal status, and histological type, were analyzed. To assess differences in these characteristics, the chi-square test was used, and Fisher’s exact test was applied when significant differences were observed.

Furthermore, the normality of the distribution of the quantitative variables was verified. For results with a normal distribution, the T-test was applied. Conversely, when the data did not follow a normal distribution, the Mann–Whitney test was used. The results were expressed as mean ± SD, and statistical significance was considered at *p* < 0.05.

## 3. Results

### 3.1. Analysis of Tumor Characteristics: Histological Grade, Tumor Stage, Nodal Status, Histological Type, and Immune Checkpoint Expression

The study included 40 patients with breast carcinoma; of these, 23 were assigned to luminal A and 17 to luminal B. The demographic and clinical details of the participants are presented in Table 1.

The analysis of the patients’ age did not reveal statistically significant differences between the luminal A and luminal B groups (Table 1). This uniformity in age range helps minimize the potential impact of this variable on the analysis of the results. Furthermore, the tumors were classified according to histological grade, tumor stage, nodal status, and histological type, as previously mentioned. In this context, statistically significant differences were only found in the distribution of tumor grades between the luminal A and luminal B groups, with a predominance of grade 1 tumors in the luminal A patients (*p* = 0.023) and grade 2 in the luminal B group (*p* = 0.018) (Table 1). This difference can be explained by the intrinsic biological characteristics of each subtype of breast cancer. Luminal B tumors are generally more aggressive and exhibit a higher cell proliferation rate than luminal A tumors, resulting in a greater frequency of grade 2 tumors in the former group. Additionally, luminal B tumors tend to express higher levels of proliferation markers, such as Ki-67, reflecting greater aggressiveness and justifying the observed differences in tumor grade distribution between the two subtypes.

On the other hand, no differences with statistical significance were found between the groups in terms of nodal status, histological type, or tumor size, suggesting a comparable distribution of nodal involvement across the tumor subtypes studied (Table 1). This uniformity reinforces the need to consider multiple variables to evaluate disease progression more comprehensively and effectively.

**Figure 3:**
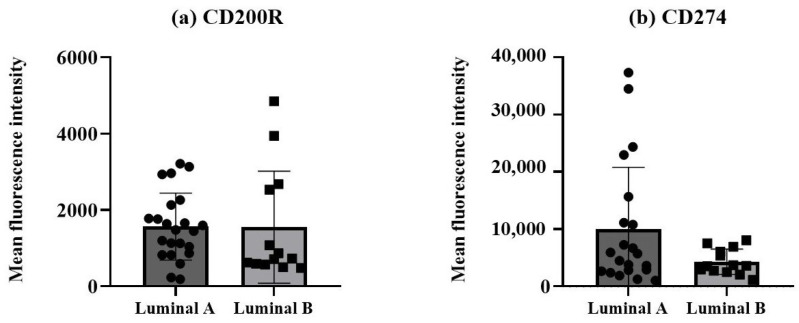
Expression of CD200R and CD274 in non-hematopoietic cells/tumor cells. Protein expressions of CD200R (**a**) and CD274 (**b**) were measured as mean fluorescence intensity (MFI). Data expressed as median and interquartile range, and statistical comparisons were performed using the Mann–Whitney non-parametric test.

The protein expression levels of the two immune checkpoint molecules, CD200R and CD274, were measured as mean fluorescence intensity (MFI) in non-hematopoietic cells, identified by the expression of cytokeratin 18 and CD326 (EpCAM) and dim/negative expression of CD45, from breast cancer biopsies. A tendency for a higher CD274 expression was observed in the non-hematopoietic cells from the luminal A tumors compared to the luminal B tumors (Figure 3b), although it did not reach statistical significance.

### 3.2. Immune Cells in the Tumor Microenvironment of Luminal Breast Cancer

To assess the TME of luminal A and B breast cancer, the tumor samples were analyzed using flow cytometry, as previously described. Although the percentage of monocytes in the two tumor subtypes was not statistically different, in the luminal A subtype the presence of monocytes in the TME was only observed in 65% of the samples, while in the luminal B subtype, it was possible to identify monocytes in 82% of the samples.

These findings indicate that, compared to luminal A, luminal B tumors tend to present a decreased percentage of monocytes and NK cells (Table 3).

### 3.3. Expression of CD200R, CD206, and CD274 in Monocytes/Macrophages

When analyzing the phenotypic characteristics of monocytes/macrophages, the luminal A group showed a tendency to exhibit a higher percentage of cells expressing CD206, although this difference did not reach statistical significance. Similarly, while the luminal A group showed higher levels of CD274 and an increased expression of CD200R, these differences did not reach statistical significance (Table 4).

### 3.4. Analysis of T Lymphocytes Present in the Tumor Microenvironment

To investigate whether the distribution of T lymphocytes and their subpopulations in the TME differs between luminal subtypes, a comprehensive analysis of the major subpopulations of T cells was conducted. Our results demonstrated that luminal B tumors presented a significant increase in the percentage of CD4^+^ T cells (*p* < 0.05). Conversely, while the luminal A tumors showed a higher percentage of γδ, CD8^+^, and CD4^−^CD8^−^ γδ^−^ T cells, these differences did not reach statistical significance, suggesting a trend toward an increased proportion of T cells with the potential to have cytotoxic activity (Table 5).

**Figure 4:**
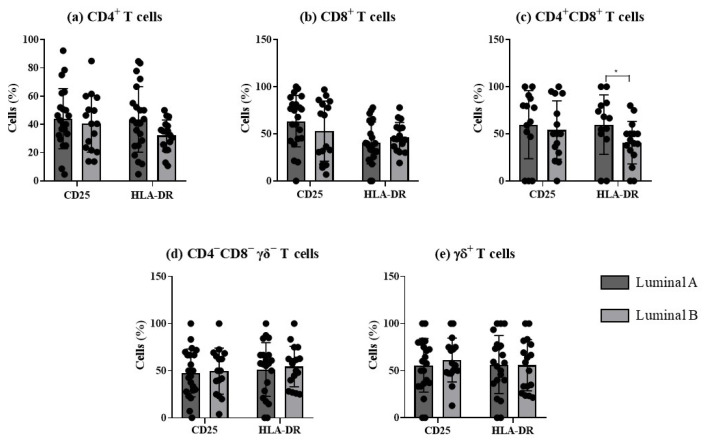
Analysis of T cells’ activation profile in luminal A and luminal B breast cancer subtypes. The percentage of T cells expressing CD25 and HLA-DR was evaluated in CD4^+^ T (**a**), CD8^+^ T (**b**), CD4^+^CD8^+^ T (**c**), CD4^−^CD8^−^ γδ^−^ T (**d**), and γδ T cells (**e**). * *p* < 0.05 indicates a significant difference between luminal A and luminal B subtypes, as determined using Mann–Whitney non-parametric test. All results are presented as mean ± SD.

The analysis of the frequency of activated T cells, based on the expression of the early and late activation markers, CD25 and HLA-DR, respectively, revealed significant differences in HLA-DR double-positive T cells between the two luminal tumor subtypes (Figure 4).

Upon antigen challenge, naïve T cells differentiate into memory cells or effector cells. Memory cells can be classified, at least, as central memory cells or effector memory cells, depending on their circulation pattern, where the former have the ability to migrate into the secondary lymphoid organs and the latter into peripheral tissues. After identifying CD4^+^, CD8^+^, CD4^+^CD8^+^, CD4^−^CD8^−^ T, and γδ T cells, these cells were further classified based on their maturation-associated compartments as naïve, central memory, effector memory, and terminal effector cells. The memory phenotype was predominant in both breast cancer subtypes. Additionally, a trend toward a higher prevalence of effector CD8^+^ T cells in luminal A (*p* > 0.05) was observed, pointing to a more effective cytotoxic action of T cells in this cancer subtype. In luminal B tumors, the central memory compartment tends to be augmented among CD4^+^ T, CD8^+^ T, and γδ T cells (*p* > 0.05), while in the CD4^−^CD8^−^ T cell population there is a prevalence of terminal effector cells in luminal B (*p* > 0.05), compared to luminal A (Supplementary Table S1).

The distribution of T lymphocytes across their functional compartments revealed differences between luminal A and luminal B breast cancer subtypes, as shown in Supplementary Figure S1. Of note, luminal B tumors presented a higher proportion of regulatory T (Treg) and Th17 cells and a lower percentage of Th1 and follicular T (Tf) cells. The results obtained for the CD4^+^ T and CD8^+^ T cell subpopulations were analyzed in detail in the following sections.

### 3.5. T Cell Polarization in the Luminal A and Luminal B Tumor Microenvironment

**Figure 5:**
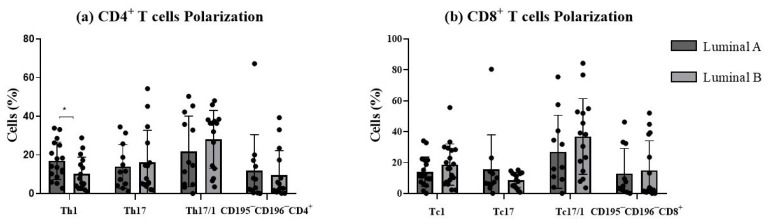
Functional phenotype analysis of T cells. The graphs display the frequencies of Th1, Th17, Th17/1, and CD195^−^CD196^−^ among CD4^+^ T cells (**a**) and Tc1, Tc17, Tc17/1, and CD195^−^CD196^−^, among CD8^+^ T cells (**b**). * *p* < 0.05 denotes statistically significant differences between tumor samples, as determined by Mann–Whitney test. Data are presented as mean ± SD.

In this study, we examined the polarization sense of tumor-infiltrating T cells according to the expression of CD195 and CD196 (Figure 5). This strategy allowed the identification of the pro-inflammatory Th1 and Tc1 cells (CD4^+^ and CD8^+^ T cells, respectively, expressing CD195 in the absence of CD196), Th17 and Tc17 (CD4^+^ and CD8^+^ T cells, respectively, expressing CD196 in the absence of CD195), and plasticity Th17/1 and Tc17/1 (CD4^+^ and CD8^+^ T cells, respectively, expressing both CD195 and CD196 markers). The remaining CD195^−^CD196^−^ T cells corresponded to those that are not polarized towards Th/c1 nor Th/c17. We decided to analyze only CD4 and CD8 T cells, since the other subpopulations were less represented and thus did not allow a clear discrimination of these functional T cell subsets.

Th1/17 and Tc1/17 were the most represented functional compartments for both luminal A and luminal B. In luminal B tumor samples, a significant decrease in the percentage of Th1 cells was observed (*p* < 0.05, Figure 5a), accompanied by a slight increase in Th17 and Th17/1 cells (*p* > 0.05, Figure 5a), compared to the luminal A tumor. No statistically significant differences were observed for CD8^+^ T cell functional subpopulations between the two tumor subtypes (Figure 5b). This suggested that luminal B tumors may exhibit a distinct T cell plasticity profile, potentially influencing their immune microenvironment.

Additionally, the activation status of these functional T cell subtypes (Supplementary Figure S2) revealed no statistically significant differences between the luminal A and B tumors.

When analyzing the distribution of polarized T cells among the maturation-associated compartments, it was observed that there was a predominance of memory cells, in both the luminal A and luminal B tumors. A significant increase in terminal effector Th1 cells (*p* < 0.05) was observed in the luminal B tumors compared to the luminal A tumors (Supplementary Table S2). Furthermore, there was a trend toward an increase in terminal effector CD195^−^CD196^−^CD8^+^ T cells in the luminal B tumors.

### 3.6. Analysis of Follicular T Cells in the Tumor Microenvironment

Additionally, follicular T cells were examined, revealing similar levels between luminal A and luminal B tumors (Supplementary Figure S3).

Regarding the activation profile of follicular T cells, no differences were observed in the percentage of CD4^+^ follicular T cells expressing the early activation marker CD25 or the later activation marker HLA-DR between luminal A and luminal B cancer (*p* > 0.05) (Supplementary Figure S4). No differences were observed in the CD8^+^ cells.

Furthermore, the distribution of follicular T lymphocytes among their maturation-associated compartments is detailed in Supplementary Table S2. The results demonstrate that both CD4^+^ and CD8^+^ terminal effector follicular T cells are increased in the luminal A group compared to the luminal B group (*p* > 0.05) (Supplementary Table S2).

### 3.7. Analysis of Regulatory T Cells in the Tumor Microenvironment

**Figure 6:**
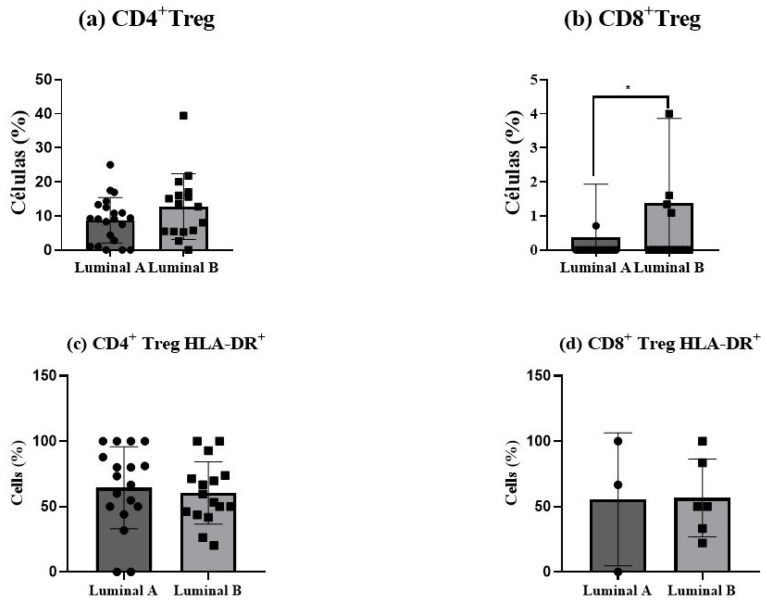
Regulatory T (Treg) cells in luminal breast cancer biopsies. The proportion of CD4^+^ Tregs was measured among tumor-infiltrating CD4^+^ T cells (**a**), and the percentage of CD8^+^ Tregs was analyzed within CD8^+^ T cells (**b**). The activation status of Treg cells was determined based on HLA-DR expression in CD4^+^ Tregs (**c**) and CD8^+^ Tregs (**d**). Data are presented as mean ± SD. * *p* < 0.05 indicates statistically significant differences between luminal A and luminal B tumors, as determined by the Mann–Whitney test.

Regulatory T (Treg) cells showed an increase in luminal B tumor samples, for both CD4^+^ T (*p* > 0.05) and CD8^+^ T cells (*p* < 0.05), as depicted in Figure 6a,b. This elevation suggests a more immunosuppressive tumor environment in luminal B tumors compared to luminal A. In turn, no differences in Treg activation status were observed (Figure 6c,d).

Interestingly, the analysis of the maturation-associated compartments of the Treg cells revealed a higher proportion of terminal effector CD4^+^ Treg and CD8^+^ Treg cells in the luminal A tumors, together with an augmented proportion of effector memory CD8^+^ Treg cells in luminal B, although it did not reach statistical significance (Supplementary Table S2).

### 3.8. Analysis of the Correlation Between the Frequency of Functional T Cell Subsets and Ki-67 Levels

**Figure 7:**
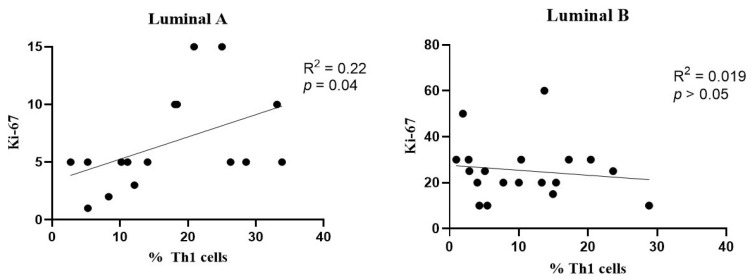
Association between the frequency of Th1 cells and Ki-67 levels. Correlation data is shown for luminal A (**left**) and luminal B (**right**) breast cancer subtypes.

A slight positive association was observed between the frequency of Th1 cells and Ki-67 levels in the luminal A breast cancer subtype (Figure 7, left), but not in the luminal B tumors (Figure 7, right).

**Figure 8:**
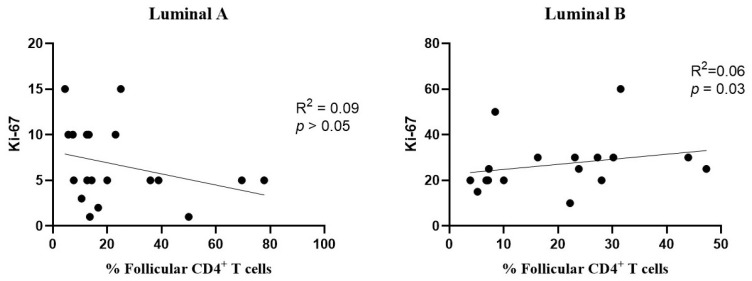
Association between the frequency of follicular T cells and Ki-67 levels. The correlation was evaluated for luminal A (**left**) and luminal B (**right**) breast cancer subtypes.

Additionally, a positive correlation was found between the levels of follicular CD4^+^ T cells and Ki-67 in the luminal B subtype (Figure 8, right), but not in luminal A (Figure 8, left).

### 3.9. Principal Component Analysis Reveals Distinct Immune Profiles in Luminal A and B Breast Cancer Subtypes

**Figure 9:**
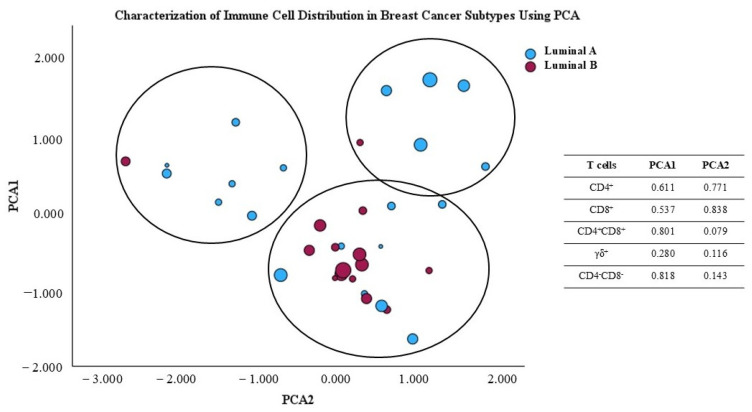
Principal component analysis (PCA) scores plot of T cell subpopulation distribution in the luminal A and luminal B tumor microenvironment.

The principal component analysis (PCA) scatter plot shown in Figure 9 illustrates the distribution of breast cancer samples from luminal A and luminal B subtypes, based on their variability as represented in the first two principal component axes. factor. This analysis was conducted to reduce the dimensionality of the immune data and facilitate visualization of the differences between the two groups. The principal factors were obtained from the percentage of major T cell subpopulations, namely CD4^+^, CD8^+^, γδ, CD4^+^CD8^+^, and CD4^−^CD8^−^ γδ^−^ T cells, present in the TME. Additionally, symbol size represents the Ki-67 value for the analyzed samples. Using PCA, we observe that almost all luminal B tumors are grouped together, indicating that they share immunologic characteristics, while luminal A tumors seem to display heterogeneous immunologic features. This could, potentially, contribute to heterogeneous clinical courses in luminal A patients. Interestingly, luminal A patients with higher Ki-67 seem to be clustered together (Figure 9).

The immunological differences observed in the PCA plot suggest that the two breast cancer subtypes exhibit variations in the characteristics of the TME concerning the presence of the studied T cells. This aligns with the notional knowledge that different cancer subtypes may recruit and interact with the immune system in distinct ways, which can impact prognosis and treatment response.

## 4. Discussion

The relationship between cancer cells and the immune system plays a pivotal role in tumor development and metastasis, significantly influencing cancer development, patient prognosis, clinical parameters, and treatment responses [2]. Immune surveillance is essential for detecting and eliminating tumor cells, while immune evasion mechanisms allow cancer cells to escape the action of the immune system. This balance is crucial in the TME, where immune cells play a significant role [2,3].

In this study, we found that while the luminal A and luminal B subtypes of breast cancer differed significantly in histological grade, these differences can have a substantial impact on the TME. Luminal B tumors, which typically have a higher histological grade and faster proliferation rate, may lead to changes in the TME, contributing to their more aggressive nature. In contrast, luminal A tumors, which are generally lower grade and slower progressing, present a different TME. Recent studies, including one by Zhou et al. (2024) [28], emphasize the critical role of histological grade and tumor stage in shaping the TME, suggesting that histological features may influence the TME significantly, affecting disease progression and therapeutic outcomes.

Building on this, we explored the cellular composition of the TME in breast cancer tissue samples from patients, focusing on the infiltration of monocytes and lymphocyte subsets and their clinical implications. Previous studies suggest that lymphocyte infiltration into tumors is associated with improved prognosis in individuals with cancer [4,6]. In line with this, we conducted a study detailing the lymphocyte subsets, as well as their expression of immune checkpoint proteins (CD200R/CD200 and PD-1/PD-L1), infiltrating breast tumors.

CD200R, an inhibitory receptor expressed on various immune cells, including M2 macrophages, monocytes, and some T cells, interacts with its ligand, CD200, to suppress immune activity [19]. The CD200/CD200R pathway is particularly relevant in tumors with a high expression of CD200, aiding immune evasion by reducing T cell and macrophage activity [19]. Our findings did not show statistically significant differences between luminal A and B tumors, in relation to the expression of CD200R on non-hematopoietic cells, suggesting that CD200/CD200R-mediated immune suppression may not be a dominant mechanism in the breast cancer subtypes under study.

CD274, also known as programmed death-ligand 1 (PD-L1), binds to the PD-1 receptor on T cells, inhibiting their effector functions and allowing tumor cells to evade immune surveillance [13]. The PD-1/PD-L1 interaction is a key mechanism by which tumors escape immune responses and avoid destruction by the immune system [14]. Some cancer cells increase the expression of PD-L1, which may hinder immune responses and help these cells evade elimination [14]. Inhibiting this pathway with monoclonal antibodies, such as PD-1/PD-L1 inhibitors, represents a promising treatment strategy with positive results in various malignancies [15]. Our results did not show significant differences between breast cancer subtypes regarding the expression of PD-L1, although this expression seemed to be increased in the luminal A subtype.

Concerning T and NK cells infiltrating the tumor, our results did not reveal notable differences in the proportion of total T cells between the luminal A-like and luminal B-like breast cancer subtypes studied. Additionally, we assessed the percentage of NK cells, which are essential for the immune response against cancer cells. NK cells recognize and destroy cancer cells through different mechanisms, including the secretion of cytotoxic granules that trigger apoptosis in abnormal cells [7,8]. Interestingly, luminal B tumors displayed a lower percentage of NK cells, compared to luminal A, indicating that the NK cell-mediated immune response could be hampered in this subtype.

In turn, monocytes can contribute to tumor growth and progression [9]. These cells can polarize into either M1 or M2 macrophages; M1 macrophages are typically associated with anti-tumor immunity and M2 macrophages contribute to immune suppression and tumor progression [29]. M1 macrophages are usually recognized by the expression of CD86 and CD64 and by the production of CXCL9, which are indicative of their pro-inflammatory and anti-tumor activity. In contrast, M2 macrophages, which are associated with immune suppression, express CD206 and CD163 [30]. Wang et al. demonstrated that monocytes could secrete CXCL7, a chemokine that stimulates cancer cell migration, invasion, and metastasis, thereby promoting cancer progression [10]. In our study, the percentage of monocytes was similar between the two tumor subtypes, as well as in the proportion of monocytes expressing CD206 and PD-L1.

CD206, also known as the mannose receptor, is predominantly located on the surface of macrophages, as well as on monocytes that develop into macrophages. This receptor is associated with immune response regulation and is elevated in tumors, correlating with poorer prognosis in various cancers, including breast cancer [16]. Furthermore, CD206-expressing macrophages can secrete cytokines like IL-10 and TGF-β, which promote an immunosuppressive environment [17,18].

Tumor-infiltrating T lymphocytes are essential in mediating anti-tumor responses [6]. Herein, we observed higher frequencies of CD4^+^ T cells in the luminal B samples; on the other hand, in luminal A we found a slight increase in T cells with cytotoxic potential, namely CD8^+^, γẟ, and CD4^−^CD8^−^ γẟ^−^ T cells. CD4^+^ T lymphocytes are important for recruiting and regulating adaptive immune responses, with their subtype distribution varying throughout carcinogenesis stages, indicating their role in tumor progression [21,23]. Concerning CD4^+^CD8^+^ T cells, initially described as a developmental stage of T cells in the thymus, our study found a higher percentage of CD4^+^CD8^+^ T-expressing HLA-DR in the luminal A subtype. Recent studies indicate that these cells may play an immunomodulatory role, either suppressing the anti-tumor immune response or promoting a pro-inflammatory environment that favors tumor progression [31]. However, the literature on the impact of CD4^+^CD8^+^ T cells involved in cancer remains contradictory, with some studies pointing to a cytotoxic function of these cells that contributes to the elimination of tumor cells [32].

The memory phenotype was predominant in T cell subpopulations infiltrating breast tumors, with a trend toward higher percentages of central memory T cells in luminal B tumors. The literature suggests that a higher frequency of memory T cells is associated with favorable cancer characteristics, such as smaller tumor size, lower disease stages, and generally better prognosis [33,34]. This is consistent with the role of memory T cells in sustaining long-term immune responses against tumors.

Different memory T cell subpopulations play distinct roles in the tumor microenvironment. T cell subpopulations, including naïve and memory T cells, play distinct but complementary roles in the tumor microenvironment. Naïve T cells are critical for initiating primary immune responses, as they recognize previously unencountered tumor antigens and differentiate into effector or memory T cells [33]. Their presence may reflect the ongoing activation of the immune system in response to tumor-associated antigens.

Different memory T cell subpopulations play distinct roles in the tumor microenvironment. Among memory T cells, central memory T cells are particularly important due to their high proliferative capacity and potential to differentiate into effector cells upon re-exposure to tumor antigens, contributing to sustained and effective immune surveillance [35,36,37]. Effector memory T cells, on the other hand, have a more immediate cytotoxic capacity, directly targeting tumor cells but with a more limited lifespan compared to central memory T cells [35,36].

The presence of these T cell subpopulations in luminal B tumors may suggest a more robust immune response, potentially enhancing the immune system’s ability to control tumor growth and metastasis.

The TME is significantly influenced by the activity of CD4^+^ T helper cells, which play a critical role in orchestrating adaptive immune responses. CD4^+^ T cells contribute to the immune system’s ability to combat tumor progression through their differentiation into various subtypes, such as Th1, Th2, Th17, and regulatory T cells (Tregs), among others, each with distinct cytokine expression profiles and immune-modulatory functions [38]. Th1 cells promote anti-tumor immunity by activating macrophages and cytotoxic T cells, while Th2 and Th17 cells can create a pro-inflammatory or immunosuppressive environment, depending on the context [21,39]. The balance of these Th cell subsets within the TME shapes the immune landscape, influencing tumor growth, immune evasion, and responses to therapy.

Th1 cells are known for promoting cellular immune responses by activating macrophages and producing cytokines, such as interferon-gamma (IFN-γ) and tumor necrosis factor-alpha (TNF-α), which are essential for mediating cytotoxic immune responses [21]. We found that the luminal A tumor samples had a significantly higher frequency of Th1 cells (with an increased activation status) compared to the luminal B tumor samples. IFN-γ activates STAT1 and STAT4, promoting Th1 differentiation, while inhibiting Th2 and Th17 differentiation, with the latter being linked to tumor progression [40]. In this study, we identified a positive correlation between Th1 cell infiltration in luminal A tumors and Ki-67 expression, suggesting that as the number of Th1 cells increases, tumor cell proliferation also tends to increase; this is potentially due to an inflammatory microenvironment. However, another study reported a negative correlation between Th1 cell presence and Ki-67 levels, indicating that higher Th1 cell counts were associated with a decreased tumor proliferation rate, suggesting a protective role for Th1 cells in limiting tumor growth [39]. These contrasting findings highlight the complexity of the Th1 response in tumor progression and suggest that additional studies are needed to elucidate the function of Th1 cells in different tumor contexts.

Furthermore, recent evidence suggests that Th1 cells may also interact with cancer stem cells (CSCs) within the TME, which could modulate their effects on tumor proliferation. CSCs are known to be resistant to conventional therapies and are implicated in tumor recurrence, and it is possible that Th1 cell-mediated inflammation could either support or inhibit CSC survival, depending on the specific tumor context. The inflammatory cytokines produced by Th1 cells, particularly IFN-γ, induce tumor cell death through the activation of cytotoxic T cells and natural killer cells and promote the infiltration of immune cells that can effectively target and eliminate tumor cells [41]. On the other hand, in certain contexts, the inflammatory microenvironment created by Th1 cells could enhance tumor progression by promoting angiogenesis and the survival of CSCs, thereby potentially facilitating tumor recurrence [42]. Thus, the balance between Th1-mediated anti-tumoral immunity and the promotion of an inflammatory environment in the TME may depend on the specific characteristics of the tumor and the immune context. Therefore, a more nuanced understanding of the interplay between Th1 cells and CSCs is critical for developing targeted therapeutic strategies.

Th17 cells, in contrast, are primarily pro-inflammatory, secreting cytokines like IL-17, which can promote chronic inflammation and myeloid cell recruitment. This environment can facilitate tumor progression by supporting angiogenesis and suppressing effective immune response [43]. Luminal B tumors exhibited a trend toward a higher frequency of Th17 cells (and an increased percentage of activated Th17 cells) compared to luminal A tumors. These findings reflect the worse prognosis associated with luminal B compared to luminal A. This plasticity may favor a TME that is more conducive to disease progression. Th17 cell activation is generally associated with the secretion of cytokines that promote an immunosuppressive environment, such as IL-17 and TGF-β, which can suppress the cytotoxic activity of T cells and NK cells, promoting the evasion of immune surveillance by the tumor. Previous studies have shown that increased IL-17 in the breast cancer TME is positively correlated with more advanced disease stages, higher proliferation indices, and more aggressive molecular subtypes [14,44,45]. However, while Th17 cells are predominantly pro-tumoral, there is evidence suggesting that they can exert anti-tumoral effects in specific contexts. For instance, IL-17 may activate tumor-associated macrophages (TAMs), promoting an immune response that could limit tumor growth in certain circumstances [46].

Treg cells play a crucial role, which is particularly relevant in breast cancer. However, the frequency of these cells across different breast cancer subtypes and their prognostic value remain controversial, with studies showing conflicting results [47,48,49,50]. Previous studies suggest that Treg infiltration is linked to specific breast cancer subtypes with more aggressive characteristics, such as high histological grade and invasive properties [51]. However, data from The Cancer Genome Atlas indicate that Treg abundance in early-stage tumors is not correlated with types of breast cancer, AJCC staging, or pathological grade, highlighting the complexity of this relationship [52]. Furthermore, Treg depletion in primary tumors has been associated with a robust anti-tumor response [53]. Treg cells are predominantly immunosuppressive, suppressing the activation and effector function of other immune cells, such as cytotoxic T cells and natural killer (NK) cells, thus promoting tumor immune evasion [48]. They exert their regulatory function by secreting immunosuppressive cytokines, such as TGF-β and IL-10, which dampen the immune response within the tumor microenvironment (TME) [20,48]. Additionally, Tregs can recruit other immunosuppressive cells, such as myeloid-derived suppressor cells (MDSCs), to further enhance immune evasion and promote tumor progression [48]. The increased Treg cells in the luminal B tumors observed in this study suggest a more immunosuppressive microenvironment that may facilitate tumor progression. Although some studies link Treg infiltration to better prognosis, others indicate decreased survival in patients with higher Treg accumulation [47,48,49,50]. This ambiguity may be attributed to a differential expression of immunosuppressive molecules and functional heterogeneity among Tregs from different patients [54].

Follicular T cells have an important role in regulating germinal center reactions and are less studied in the context of the breast cancer TME [55,56]. Previous studies have associated these cells with anti-tumor effects, including increased secretion of effector molecules such as IFN-γ and TNF-α [57]. Active follicular T cell responses in the TME may exert immune pressure against tumors, enhancing the production of tumor-specific antibodies [58]. In the luminal B tumors, we observed a positive correlation between the infiltration of CD4^+^ follicular T cells and the proliferation index Ki-67. This association suggests that an increased tumor cell proliferation may correspond to a greater presence of CD4^+^ follicular T cells in luminal B tumors. This finding indicates a potentially complex function of CD4^+^ follicular T cells within the TME of breast cancer. While these cells may contribute to anti-tumor immune responses by enhancing immune surveillance and antibody production, their association with elevated Ki-67 levels in luminal B tumors could reflect an environment of increased cellular activity, potentially supporting both tumor proliferation and immune responses.

Overall, our results suggest that the reduction in NK cells, CD4^−^CD8^−^γδ^−^, γδ T cells, and Th1 cells in luminal B tumors, together with an increase in Tregs and CD196-expressing cells (Th17, Th1/17, Tc17, and Tc1/17), may contribute to an immunosuppressive environment favoring tumor progression. This study underscores the importance of thoroughly characterizing the TME to comprehend the intricate interactions between immune and cancer cells.

Despite the observed trends, it is crucial to recognize the constraints of this research, including sample size and inherent variability in the TME, which limits definitive conclusions. Expanding sample size will help to validate observed trends and explore their clinical implications, leading to a deeper understanding of TME interactions.

Finally, the complex relationship between the TME and tumor progression highlights the need for a therapeutic approach that considers not only the intrinsic characteristics of tumor cells but also the role of immune cells and other TME components. Integrating immunological data with clinical and molecular characteristics can provide valuable insights for personalizing therapeutic strategies and potentially improving clinical outcomes for breast cancer patients.

## 5. Conclusions

In summary, this study demonstrates the presence of a different composition of the TME between luminal A and B breast cancer subtypes. Namely, it shows that luminal B tumors exhibit a significantly higher percentage of CD4^+^ T cells, along with increased frequencies of Treg cells, particularly of CD8+ Tregs. On the other hand, in this subtype we observed lower levels of NK cells, and of Th1 cells, meaning that the luminal B tumor have a more immunosuppressive TME, which may influence tumor behavior and patient prognosis.

These findings highlight the importance of a detailed characterization of the TME, revealing the diversity of immune cell populations and their impact on treatment response. A deeper understanding of these dynamics could inform the development of novel therapeutic strategies targeting specific components of the TME, contributing to more personalized treatment approaches. Additional research is needed in larger cohorts to elucidate the mechanisms and immunological differences between the molecular subtypes of breast cancer.

## Figures and Tables

**Table 1:** Comparison of the demographic and clinical characteristics of patients with luminal A and luminal B tumor subtypes.

Variable	All Patients (*n* = 40)	Luminal A (*n* = 23)	Luminal B (*n* = 17)	*p*-Value
**Age**				
Mean ± SD	59 ± 7	58 ± 7	60 ± 6	0.28
Age range	36–69	36–69	51–69	
**Histological grade, *n* (%)**				
1	14 (36%)	13 (59%)	1 (6%)	0.023
2	22 (56%)	8 (36%)	14 (82%)	0.018
3	3 (8%)	1 (5%)	2 (12%)	1.0
**Tumor size (mm), *n* (%)**				
0–10	4 (16%)	2 (14%)	2 (79%)	0.26
10–20	16 (64%)	10 (71%)	6 (43%)	0.38
20–30	4 (16%)	1 (7%)	3 (21%)	0.17
>30	1 (4%)	1 (7%)	0 (0%)	0.36
**Status Nodal, *n* (%)**				
N0	16 (68%)	8 (73%)	8 (64%)	0.75
N1	7 (32%)	3 (27%)	4 (33%)	0.48
**Cut-Off Ki-67, *n* (%)**				
<20%	27 (68%)	23 (100%) *	4 (24%) *	0.001
20–40%	11 (28%)	0 (0%) *	11 (65%) *	0.001
>40%	2 (5%)	0 (0%)	2 (12%)	0.092
**Histological Type, *n* (%)**				
Invasive Carcinoma	36 (90)	20 (86%)	15 (94%)	0.46
Lobular Carcinoma	2 (5)	2 (9%)	-	0.21
Mixed mucinous Carcinoma	2 (5%)	1 (5%)	1 (6%)	0.83

* *p* < 0.05 indicates a significant difference between luminal A and luminal B subtypes, as determined using Mann–Whitney non-parametric test. All results are presented as mean ± SD.

**Table 2:** Monoclonal antibody panel used for the identification of monocytes/macrophages and T cells, indicating their corresponding fluorochrome, clone, and commercial source.

Fluorochrome	Tube 1			Tube 2			Tube 3		
	Antibody	Clone	Commercial Source	Antibody	Clone	Commercial Source	Antibody	Clone	Commercial Source
FITC	HLA-DR	L243	BD Biosciences	TCRγδ	IMMU510	Beckman Coulter	Cytokeratin 18	Ks18.04	Cytognos
PE	CD200R	OX-108	Biolegend	CD4	SK3	BD Bioscience	Epcam	EBA-1	BD Biosciences
PerCP-Cy5.5	CD206	15-2	Biolegend	CD196	11A9	BD Pharmingen	-	-	-
PE-Cy7	CD16	3G8	BD Pharmingen	CD127	HIL-7R-M21	BD Pharmingen	-	-	-
APC	CD33	P67.6	BD Biosciences	CD25	2A3	BD Bioscience	CD200	MRC OX-104	BD Bioscience
APC R700	-	-	-	CD27	M-T271	BD Horizon			
APC-H7	-	-	-	CD8	SK1	BD Bioscience	-	-	-
PB	CD274	29E.2A3	Biolegend	CD185	RF8B2	BD Horizon	-	-	-
PO	CD45	2D1	BD Biosciences	HLA-DR	G46-6	BD Horizon	CD45	2D1	BD Biosciences
BV605	-	-	-	CD3	SK7	BD Horizon	-	-	-
BV711	-	-	-	CD195	2D7/CCR5	BD Horizon	-	-	-
BV786	-	-	-	CD45RA	HI100	BD Horizon	-	-	-

Abbreviations: APC—Allophycocyanin; APC-H7—Allophycocyanin-hilite 7; BV—Brilliant violet; FITC—Fluorescein isothiocyanate; PB—Pacific blue; PE—Phycoerythrin; PerCP-Cy5.5—Peridinin chlorophyll protein cyanine 5.5; PE-Cy7—Phycoerythrin Cyanine 7; PO—Pacific orange. Commercial source: BD Biosciences—Becton Dickinson Biosciences, San Jose, CA, USA; BD Horizon—Franklin Lakes, NJ, USA; BD Pharmingen, San Diego, CA, USA; Beckman Coulter—Miami, FL, USA; Biolegend—San Diego, CA, USA.

**Table 3:** Percentages of non-hematopoietic cells and immune cells infiltrating the tumor, including T cells, NK cells, and monocytes/macrophages, in luminal A and luminal B breast tumors.

Cell Types	Luminal A	Luminal B	*p*-Value
**Non-hematopoietic cells/Tumor cells**	65 ± 22	72 ± 15	0.32
**Hematopoietic cells/Immune infiltrate**	31 ± 20	35 ± 29	0.55
**Monocytes/Macrophages**	3.04 ± 2.94	1.28 ± 1.53	0.06
**Lymphocytes**	30 ± 20	35 ± 29	0.61
**NK cells** (within the immune infiltrate)	1.25 ± 1.42	0.50 ± 0.47	0.05
NK (within lymphocytes)	5.49 ± 5.94	3.15 ± 2.64	0.13
**T cells** (within the immune infiltrate)	26 ± 18	36 ± 30	0.19
T cells (within lymphocytes)	95 ± 5.94	97 ± 2.73	0.15

All results are expressed as mean ± SD, and statistical comparisons were performed using the Mann–Whitney non-parametric test.

**Table 4:** Percentage of monocytes/macrophages expressing CD206 and CD274 in luminal A and luminal B tumor subtypes, and protein expression levels of CD200R (measured as mean fluorescence intensity, MFI) in monocytes/macrophages.

Monocytes/Macrophages	Luminal A	Luminal B	*p*-Value
CD206^+^	71 ± 6.81	55 ± 9.53	0.20
CD274^+^	70 ± 7.50	58 ± 11.97	0.34
MFI of 200R	37,672 ± 47,272	23,484 ± 28,067	0.30

All results are expressed as mean ± SD, and statistical comparisons were performed using the Mann–Whitney non-parametric test.

**Table 5:** Percentage of T cell subpopulations (measured within T cells) in luminal A and luminal B breast cancer subtypes.

Cell Types	Molecular Subtype of Breast Cancer		*p*-Value
	Luminal A	Luminal B	
CD4^+^	37 ± 3.98	52 ± 2.34 *	0.0052
CD8^+^	36 ± 4.70	32 ± 1.70	0.99
CD4^+^CD8^+^	1.46 ± 0.39	2.81 ± 0.85	0.19
CD4^−^CD8^−^ γδ^−^	17 ± 4.15	8.88 ± 1.88	0.34
γδ	8.76 ± 2.27	4.47 ± 0.79	0.69

* *p* < 0.05 indicates a significant difference between luminal A and luminal B subtypes, as determined using Mann–Whitney non-parametric test. All results are presented as mean ± SD.

## Data Availability

Dataset available on request from the authors.

## Supplementary Materials

The following supporting information can be downloaded at: https://www.mdpi.com/article/10.3390/biom15010078/s1, Supplementary Figure S1: Distribution of T lymphocyte subpopulations in luminal A and luminal B tumors; distribution of T cells by their major subpopulations: CD4^+^ T, CD8^+^ T, γδ T, CD4^+^CD8^+^ T, and CD4^−^CD8^−^ γδ^−^ T cells; and distribution of CD4^+^ T and CD8^+^ T cells by their functional subsets, in breast cancer samples from luminal A and luminal B subtypes, Supplementary Figure S2: Percentage of activated T cells from luminal A and luminal B breast cancer subtypes. The percentage of activated T cells was assessed in (a) Th1, (b) Th17, (c) Th17/1, and (d) CD195^−^CD196^−^CD4^+^ T cells and (e) Tc1, (f) Tc17, (g) Tc17/1, and (h) CD195^−^CD196^−^CD8^+^ T cells. The percentage of activated cells was evaluated based on the early (CD25) and late (HLA-DR) activation markers’ expression. All results are expressed as mean ± SD, and statistical comparisons were performed using the Mann–Whitney non-parametric test, Supplementary Figure S3: Percentage of tumor-infiltrating follicular T cells. The percentage of follicular T cells was evaluated within CD4^+^ T (a) and CD8^+^ T (b) cells. All results are expressed as mean ± SD, and statistical comparisons were performed using the Mann–Whitney non-parametric test, Supplementary Figure S4: Activation status of follicular T cells. The activation was evaluated based on the expression of CD25 and HLA-DR, in CD4^+^ (a,b) and CD8^+^ (c,d) follicular T cells. All results are expressed as mean ± SD, and statistical comparisons were performed using the Mann–Whitney non-parametric test, Supplementary Table S1: Distribution of T lymphocytes within each functional compartment (naïve, central memory, effector memory, and terminal effector) in CD4^+^ T cells, CD8^+^ T cells, γδ^+^ T cells, CD4^+^CD8^+^ (double-positive) T cells, and CD4^−^CD8^−^γδ^−^ T cells (double-negative T cells), Supplementary Table S2: Characterization of the T cells present in the immune cell tumor infiltrate. T cells were characterized according to their function in Th1, Tc1, Th17, Tc17, Th1/17, Tc1/17, and CD195^−^CD196^−^ CD4^+^ T cells and CD195^−^CD196^−^ CD8^+^ T cells. Each one of the above-defined cell populations was further divided into its maturation-associated compartment: naïve, central memory, effector memory, and terminal effector T cells.
