# Supplementary material for: Early-Stage Luminal B-like Breast Cancer Exhibits a More Immunosuppressive Tumor Microenvironment than Luminal A-like Breast Cancer
Source: Biomolecules. 2025 Jan 7;15(1):78. doi: 10.3390/biom15010078 (PMC11763923; doi:10.3390/biom15010078)
Supplement: Supplementary file 1 [file biomolecules-15-00078-s001.zip › biomolecules-3365344-supplementary.pdf]

Supplementary Table S1. Distribution of T lymphocytes within each functional compartment (naïve, central memory, effector memory, and terminal effector) in CD4⁺ T cells, CD8⁺ T cells, γδ⁺ T cells), CD4⁺CD8⁺ (double-positive) T cells, and CD4⁻CD8⁻γδ⁻ T cells (double-negative T cells).

T cells	Maturation-associated Compartments	Luminal A	Luminal B	*p*-value
$CD4^+$ T cells	Naïve	7.80 ± 13	6.08 ± 9.19	0.98
	Central Memory	38 ± 22	47 ± 16	0.12
	Effector Memory	46 ±24	42 ± 16	0.73
	Terminal Effector	6.80 ± 17	4.42 ± 12	0.35
$CD8^+$ T cells	Naïve	17 ± 19	7.35 ± 11	0.25
	Central Memory	39 ± 27	49 ± 20	0.29
	Effector Memory	27 ± 26	36 ± 24	0.25
	Terminal Effector	18 ± 29	7.45 ± 8.28	0.95
$CD4^+CD8^+$ T cells	Naïve	8.92 ± 11	11 ± 8.10	0.49
	Central Memory	63 ± 36	61 ± 29	0.89
	Effector Memory	15 ± 16	19 ± 21	0.77
	Terminal Effector	11 ± 17	8.60 ± 21	0.88
$CD4^-CD8^-$ $\gamma\delta^-$ T cells	Naïve	3.99 ± 11	5.34 ± 27	0.25
	Central Memory	20 ± 36	16 ± 32	0.42
	Effector Memory	55 ± 16	43 ± 26	0.31
	Terminal Effector	20 ± 17	36 ± 24	0.29
γδ T cells	Naïve	7.10 ± 12	6.99 ± 7.21	0.37
	Central Memory	23 ± 31	27 ± 21	0.42
	Effector Memory	51 ± 27	42 ± 29	0.39
	Terminal Effector	18 ± 21	24 ± 23	0.5

All results are expressed as mean ± SD, and statistical comparisons were performed using the Mann-Whitney non-parametric test.

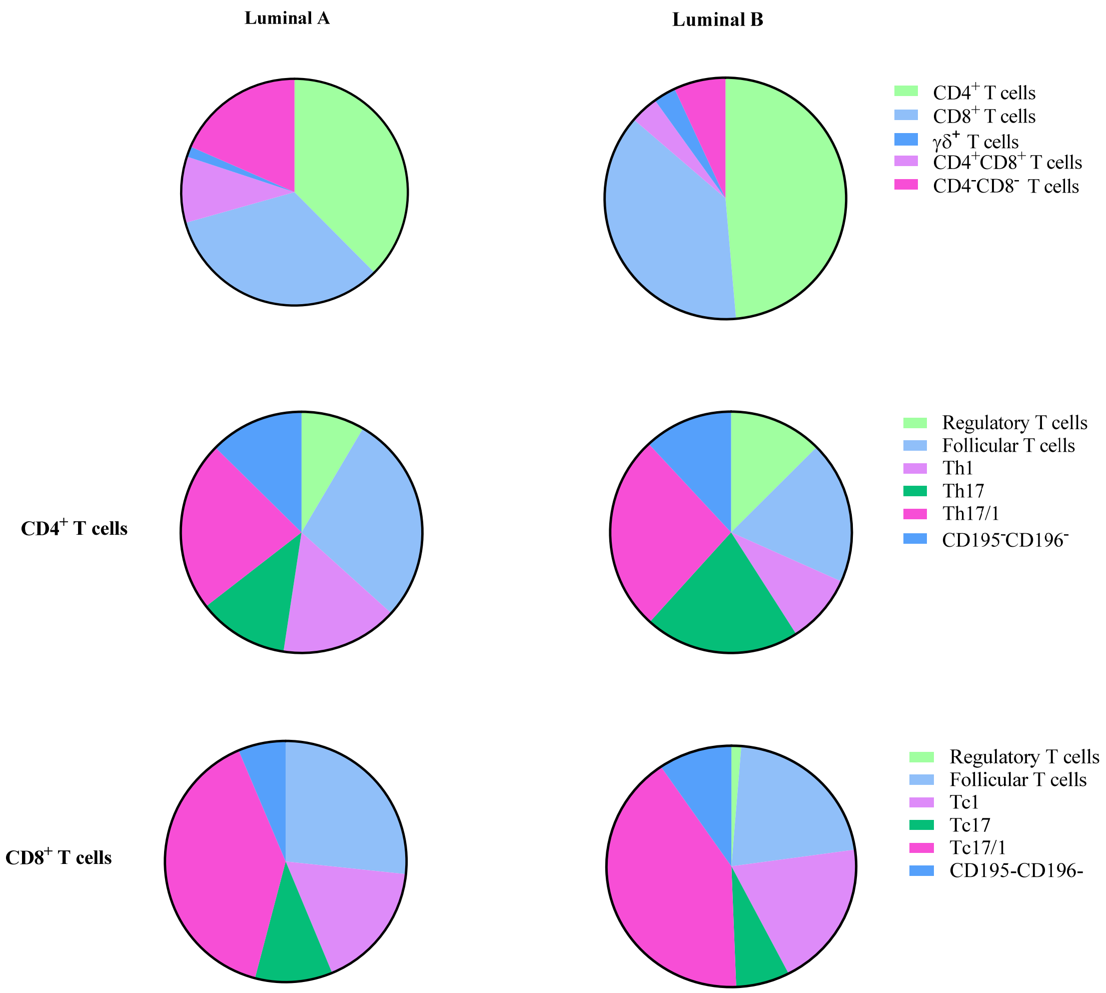

Supplementary Figure S1. Distribution of T lymphocyte subpopulations in luminal A and luminal B tumors. Distribution of T cells by their major subpopulations: $CD4^+$ T, $CD8^+$ T, $\gamma\delta$ T, $CD4^+CD8^+$ T, $CD4^-CD8^-$ $\gamma\delta^-$ T cells; and distribution of $CD4^+$ T and $CD8^+$ T cells by their functional subsets, in breast cancer samples from luminal A and luminal B subtypes.

Supplementary Table S2. Characterization of the T cells present in the immune cell tumor infiltrate. T cells were characterized, according to their function, in Th1, Tc1, Th17, Tc17, Th1/17, Tc1/17 and CD195$^-$CD196$^-$ CD4$^+$ T cells and CD195$^-$CD196$^-$ CD8$^+$ T cells. Each one of the above defined cell populations were further divided into their maturation-associated compartment: naïve, central memory, effector memory, and terminal effector T cells.

**Maturation-associated Compartments**	**CD4$^+$ T cells**	**CD4**			**CD8$^+$ T cells**	**CD8**		
		Luminal A	Luminal B	*p*-value		Luminal A	Luminal B	*p*-value
Naïve	Th1	10 ± 30	2.46 ± 4,39	0.87	Tc1	8.41 ± 20	3.98±6,40	0.76
Central Memory		38 ± 26	31 ± 39	0.41		43 ± 31	30± 26	0.29
Effector Memory		51 ± 27	63 ± 30	0.26		43 ± 28	58 ± 26	0.29
Terminal Effector		0.65 ± 2.15	4.12±6.28*	0.046		5.61 ± 8.78	6.41 ± 8.13	0.81
Naïve	Th17	14 ± 29	8.7 ± 21	0.75	Tc17	12 ± 23	12 ± 13	0.67
Central Memory		54 ± 34	51 ± 26	0.88		46 ± 33	51 ± 32	0.76
Effector Memory		33 ± 32	40 ± 31	0.49		35 ± 31	25 ± 15	0.72
Terminal Effector		0.1 ± 0,43	0.8 ± 11	0.74		6.8 ± 11	12 ± 18	0.52
Naïve	Th17/1	5.60 ± 5,55	7,6 ± 13	0.8	Tc17/1	20 ± 40	10 ± 16	0.59
Central Memory		60 ± 24	48 ± 19	0.27		53 ± 32	55 ± 25	0.87
Effector Memory		33 ± 21	43 ± 25	0.28		19 ± 12	31 ± 18	0.28
Terminal Effector		1.5 ± 4,29	1.6 ± 3.79	0.32		8.6 ± 12	3.7 ± 6.19	0.44
Naïve	CD195$^-$ CD196$^-$ *CD4$^+$ T cells*	9.27 ± 22	4.13± 11	0.58	CD195$^-$ CD196$^-$ *CD8$^+$ T cells*	7.09 ± 18	13 ± 11	0.09
Central Memory		56 ± 39	48 ± 35	0.54		55 ± 38	50 ± 15	0.49
Effector Memory		34 ± 39	48 ± 35	0.37		33 ± 37	28 ± 9.38	0.54
Terminal Effector		0.0 ± 0.0	0.59 ± 1.56	0.41		2.76 ± 7.00	9.49 ± 8.60	0.05
Naïve	Treg	4.7 ± 7.81	7.5 ± 14.17	0.69	Treg	17 ± 18	19 ± 33	0.75
Central Memory		79 ± 19	72 ± 17	0.18		39 ± 27	39 ± 21	0.87
Effector Memory		16 ± 14	21 ± 17	0.63		27 ± 26	40 ± 11	0.16
Terminal Effector		0.3 ± 1.1	0.2 ± 0.5	0.59		18 ± 29	2.1 ± 3.67	0.29
Naïve	Follicular	2.5 ± 3.77	6.8 ± 6.66	0.15	Follicular	9.43 ± 21	6.6 ± 8.72	0.82
Central Memory		47 ± 37	71 ± 23	0.12		49 ± 42	56 ± 30	0.72
Effector Memory		28 ± 36	22 ± 24	0.9		23 ± 31	29 ± 28	0.39

Terminal Effector		22 ± 40	0.2 ± 0.55	0.07		16 ± 28	8.7 ± 15	0.94

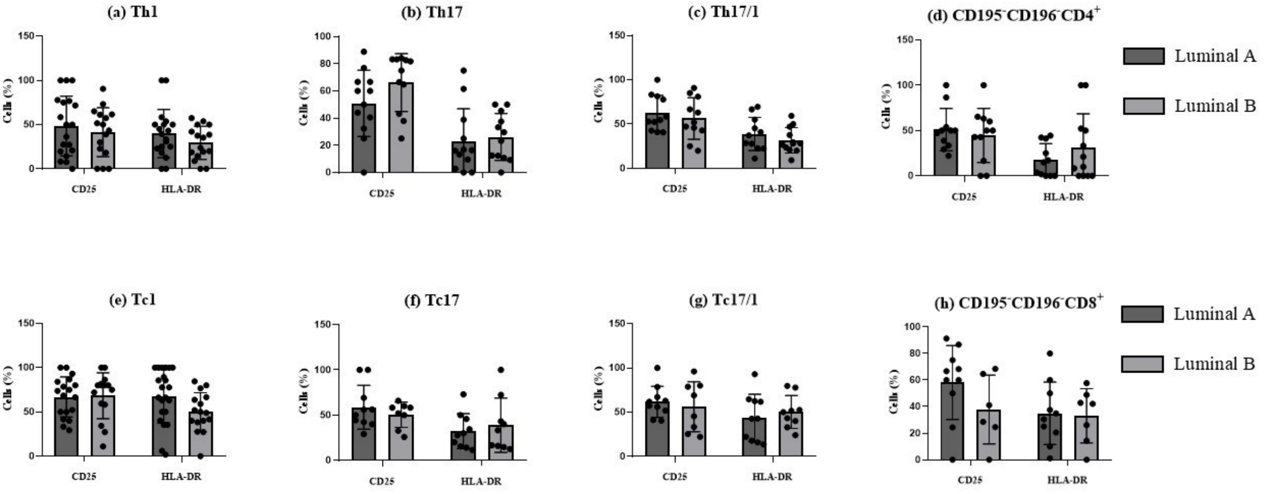

Supplementary Figure S2. Percentage of activated T cells from luminal A and luminal B breast cancer subtypes. The percentage of activated T cells was assessed in (a) Th1, (b) Th17, (c) Th17/1, (d) $CD195^{-}CD196^{-}CD4^{+}$ T cells; and (e) Tc1, (f) Tc17, (g) Tc17/1, and (h) $CD195^{-}CD196^{-}CD8+$ T cells. The percentage of activated cells was evaluated based on the early (CD25) and late (HLA-DR) activation markers' expression. All results are expressed as mean ± SD, and statistical comparisons were performed using the Mann-Whitney non-parametric test.

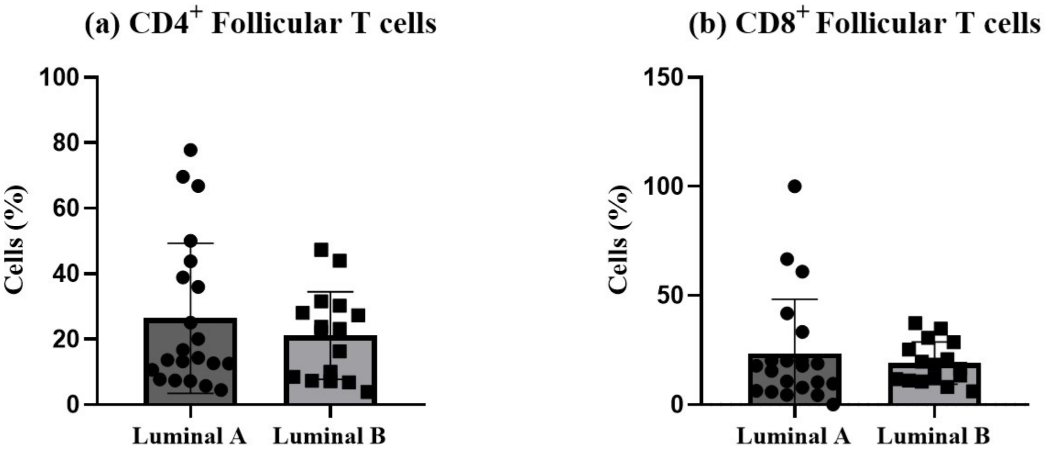

Supplementary Figure S3. Percentage of tumor-infiltrating follicular T cells. The percentage of follicular T cells was evaluated within $CD4^{+}$ T (a) and $CD8^{+}$ T (b) cells. All results are expressed as mean ± SD, and statistical comparisons were performed using the Mann-Whitney non-parametric test.

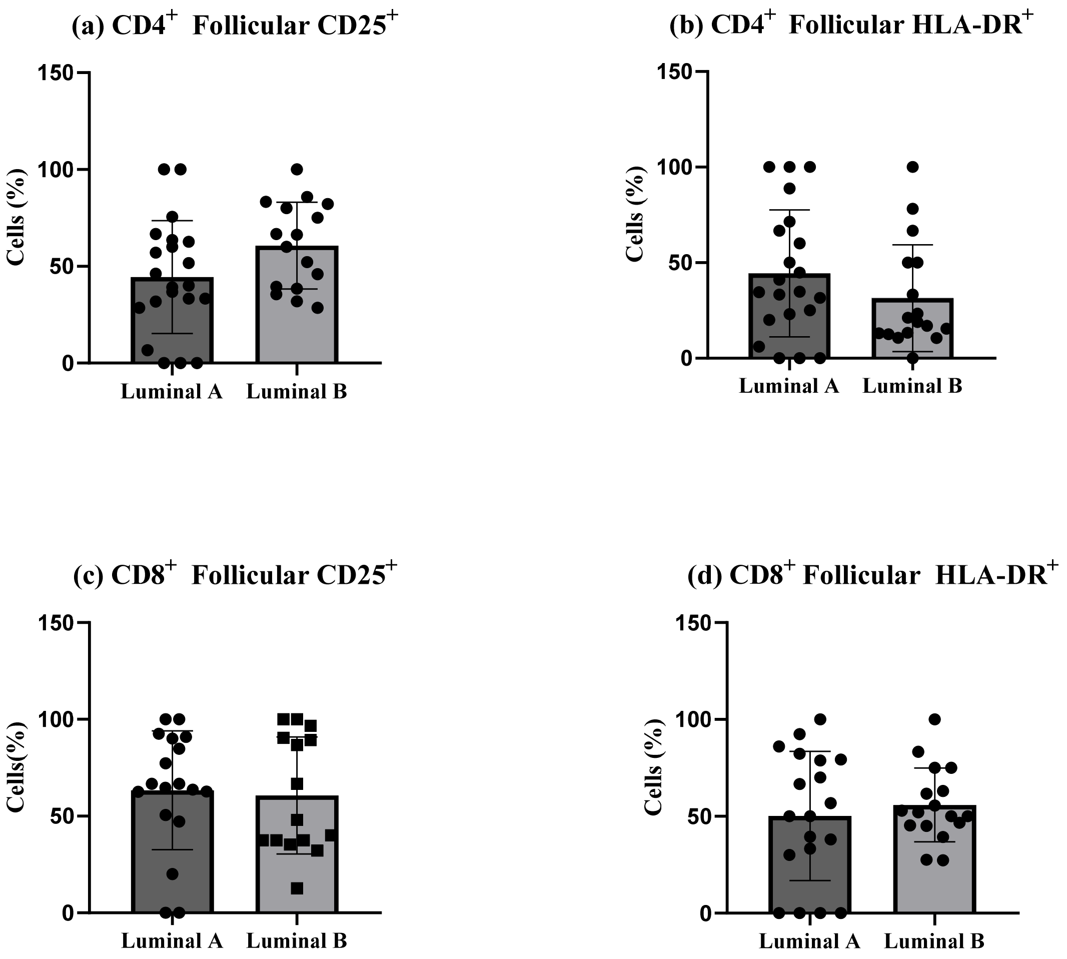

Supplementary Figure S4. Activation status of follicular T cells. The activation was evaluated based on the expression of CD25 and HLA-DR, in CD4+ (a, b) and CD8+ (c,d) follicular T cells. All results are expressed as mean ± SD, and statistical comparisons were performed using the Mann-Whitney non-parametric test.
